# Congenital Membranous Stapes Footplate Producing Episodic Pressure-Induced Perilymphatic Fistula Symptoms

**DOI:** 10.3389/fneur.2020.585747

**Published:** 2020-11-10

**Authors:** Han Matsuda, Yasuhiko Tanzawa, Tatsuro Sekine, Tomohiro Matsumura, Shiho Saito, Susumu Shindo, Shin-ichi Usami, Yasuhiro Kase, Akinori Itoh, Tetsuo Ikezono

**Affiliations:** ^1^Department of Otorhinolaryngology, Saitama Medical University, Saitama, Japan; ^2^Department of Biochemistry and Molecular Biology, Nippon Medical School, Graduate School of Medicine, Tokyo, Japan; ^3^Department of Otorhinolaryngology, Shinshu University School of Medicine, Nagano, Japan

**Keywords:** cochlin-tomoprotein, CTP, pneumolabyrinth, perilymph fistula, PLF, stapes, superior canal dehiscence, third window syndrome

## Abstract

**Introduction:** Recent third window syndrome studies have revealed that the intact bony labyrinth and differences in the stiffness of the oval and round windows are essential for proper cochlear and vestibular function. Herein we report a patient with a congenital dehiscence of the right stapes footplate. This dehiscence caused long-standing episodic pressure-induced vertigo (Hennebert sign). At the time of presentation, her increased thoracic pressure changes induced the rupture of the membranous stapes footplate. Perilymph leakage was confirmed by imaging and a biochemical test [perilymph-specific protein Cochlin-tomoprotein (CTP) detection test].

**Case Report:** A 32-year-old woman presented with a sudden onset of right-sided hearing loss and severe true rotational vertigo, which occurred immediately after nose-blowing. CT scan showed a vestibule pneumolabyrinth. Perilymphatic fistula (PLF) repair surgery was performed. During the operation, a bony defect of 0.5 mm at the center of the right stapes footplate, which was covered by a membranous tissue, and a tear was found in this anomalous membrane. A perilymph-specific protein CTP detection test was positive. The fistula in the footplate was sealed. Postoperatively, the vestibular symptoms resolved, and her hearing improved. A more detailed history revealed that, for 15 years, she experienced true rotational vertigo when she would blow her nose. After she stopped blowing her nose, she would again feel normal.

**Discussion:** There is a spectrum of anomalies that can occur in the middle ear, including the ossicles. The present case had a dehiscence of the stapes, with a small membranous layer of tissue covering a bony defect in the center of the footplate. Before her acute presentation to the hospital, this abnormal footplate with dehiscence induced pathological pressure-evoked fluid-mechanical waves in the inner ear, which resulted in Hennebert sign. When patients have susceptibility (e.g., weak structure) to rupture, such as that identified in this case, PLF can be caused by seemingly insignificant events such as nose-blowing, coughing, or straining.

**Conclusion:** This case demonstrates that PLF is a real clinical entity. Appropriate recognition and treatment of PLF can improve a patient's condition and, hence, the quality of life.

## Introduction

Third window syndrome (TWS) was first identified in patients with superior semicircular canal dehiscence (SCD) (1); now it includes cochlea-internal carotid artery dehiscence and posterior semicircular canal-jugular bulb dehiscence, cochlea-facial nerve dehiscence, and others (2). Wackym et al. classified these conditions as CT+ TWS or CT+ OCDS (i.e., third window syndrome with positive findings on CT imaging or otic capsule dehiscence syndrome with positive findings on CT imaging). The patient reported herein had no visible CT evidence of a bony dehiscence creating a third mobile window. The diagnostic findings and symptoms were similar to those of patients with CT+ TWS (3, 4).

We report a patient identified as a CT- TWS who had a bony defect of 0.5 mm at the center of the right stapes footplate which was covered by a membranous structure. This dehiscence caused a long-standing, pressure-induced vertigo. At the time of presentation, her increased thoracic pressure induced the rupture of the membranous stapes footplate, resulting in severe true rotational vertigo and hearing loss. Perilymph leakage was confirmed by imaging and a biochemical test utilizing a perilymph-specific protein Cochlin-tomoprotein (CTP) detection test.

## Case Report

A 32-year-old woman presented to another hospital with a sudden onset of right-sided hearing loss and severe true rotational vertigo, which occurred immediately after nose-blowing. She was treated with corticosteroids and bedrest for 1 week, and her vestibular symptom initially resolved. However, on the 7th day, her severe rotational vertigo recurred, and her hearing loss persisted, and she was referred to our hospital.

**Figure 1:**
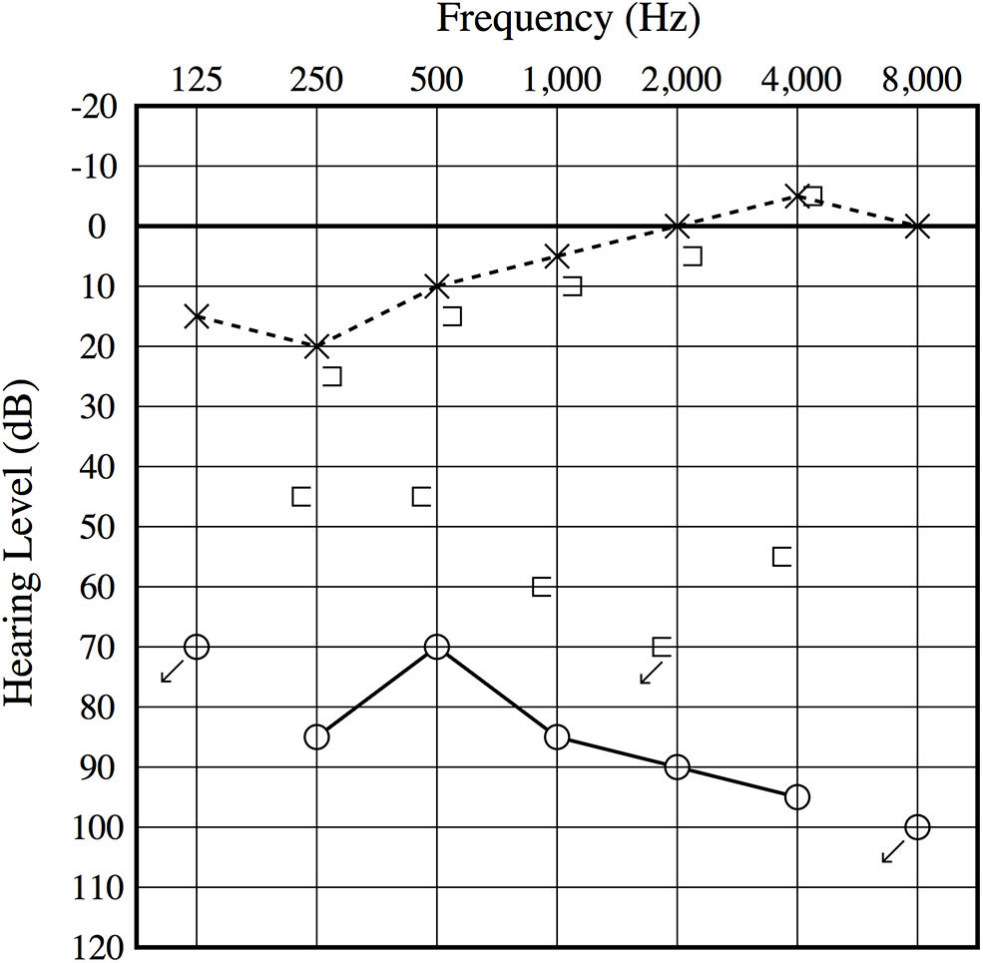
Preoperative audiogram. A severe mixed sensory and conductive hearing loss is observed on the right ear, and an air–bone gap is present at low frequencies.

**Figure 2:**
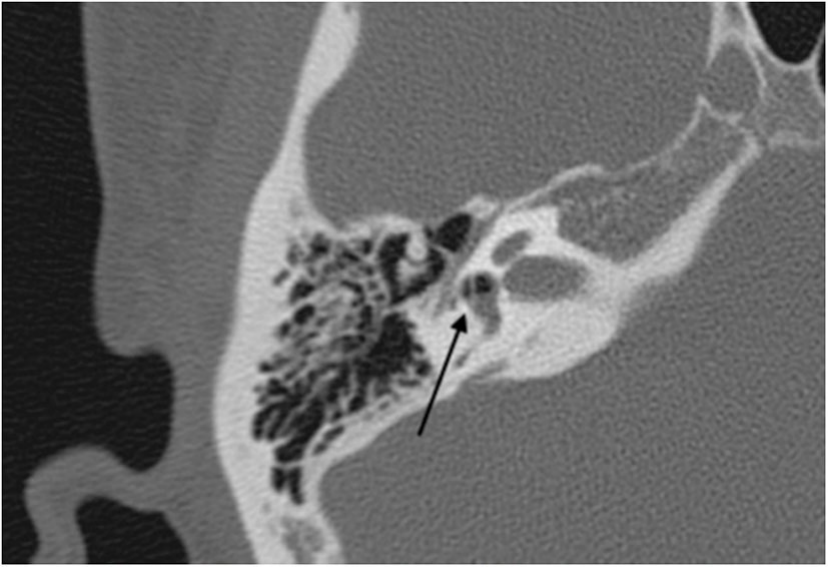
Preoperative CT scan. Air bubbles (arrow) are visible in the vestibule.

**Figure 3:**
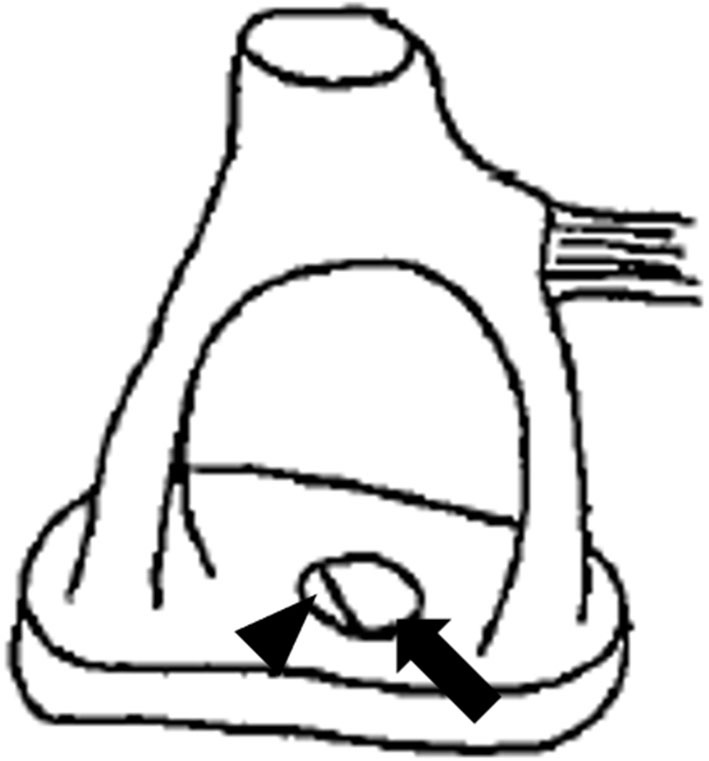
Illustration depicting the anomalous stapes footplate. The arrow illustrates the bony defect, while the arrowhead illustrates a tear in the membranous stapes footplate.

**Figure 4:**
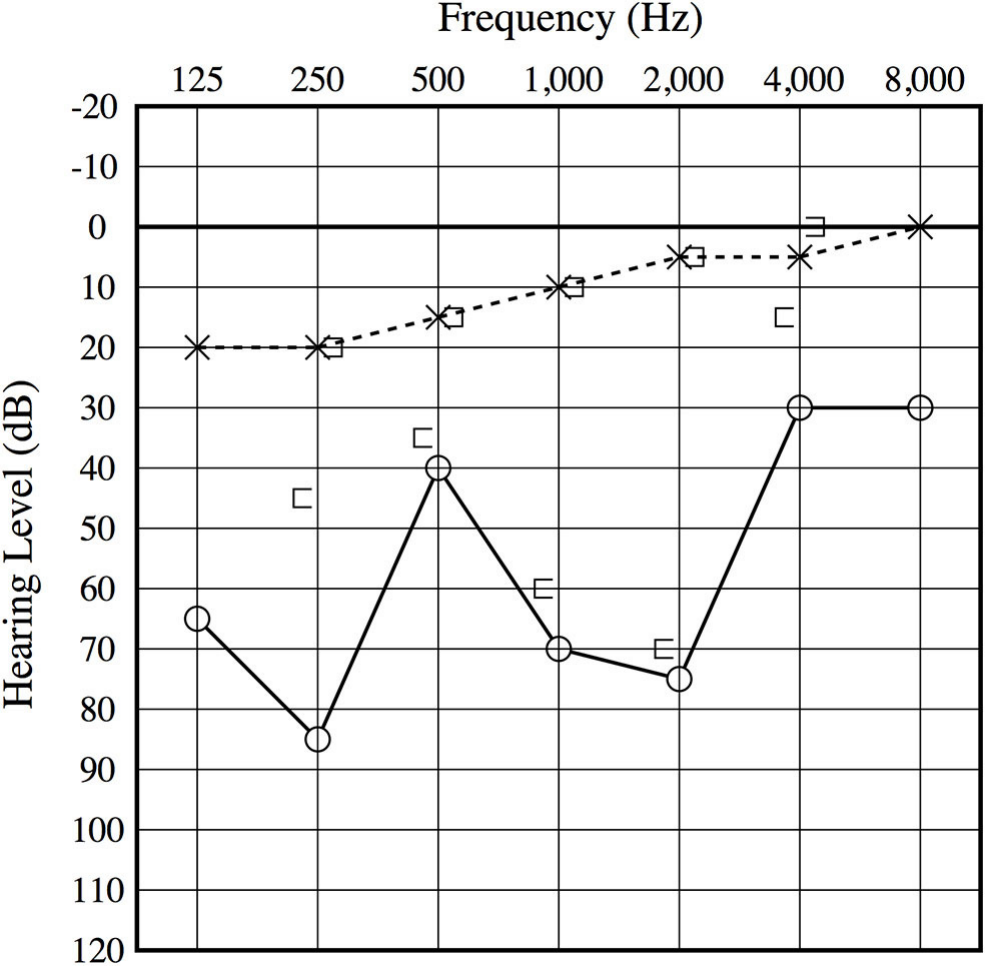
Postoperative audiogram. The 1-month postoperative audiogram had thresholds somewhat improved compared to the preoperative audiogram.

An otoscopic examination showed bilateral intact tympanic membranes. Pure tone audiometry showed a severe right-sided mixed hearing loss (Figure 1). Left-beating horizontal and rotatory nystagmus was mainly observed in the supine position with Frenzel glasses. A high-resolution temporal bone CT scan on the 10th day showed pneumolabyrinth in the right vestibule (Figure 2). She was again treated with bedrest and corticosteroids. After the conservative treatment, however, her vertigo and severe hearing loss did not resolve. Therefore, we decided to perform perilymphatic fistula (PLF) repair surgery on the 17th day. The operation was done under general anesthesia; a transcanal approach with tympanomeatal flap elevation enabled the observation of a dehiscence in the center of the stapes footplate with a bony defect 0.5 mm in diameter, which was covered by a membranous tissue, and a tear was found in this membrane (Figure 3). A small amount of perilymph leakage was observed from this tear, and middle ear lavage with 0.3 ml of saline for a CTP detection test was collected during the operation. The fistula in the footplate was sealed with connective tissue, and the round window was reinforced with connective tissue and cartilage to stabilize the labyrinth further. The vestibular symptoms and nystagmus disappeared immediately after the operation, and at her 1-month follow-up assessment, her hearing improved (Figure 4). Postoperatively, a CTP detection test revealed a concentration of 0.84 ng/ml, which is positive, with the cutoff criteria being CTP ≧ 0.8 positive, 0.8 > CTP > 0.4 intermediate, and 0.4 > CTP negative (ng/ml) (5).

A more detailed history revealed that, for 15 years, she felt vertigo as if her brain was shaken upward when she would blow her nose. True rotational vertigo would continue for 3 to 4 s. After she stopped blowing her nose, she would again feel normal. She did not hear any internal sounds such as echoing, resonant voice, pulse/heartbeat, or hearing her eyes move or blink. She did not have any history of traumatic events to her head or ears. Based upon her history, intraoperative findings, and postoperative resolution of her vestibular symptoms, the dehiscence of the stapes footplate caused pressure-induced vertigo symptoms. At 1 year after the surgery, she had no recurrence of the vestibular symptoms, and her cochlear function remained unchanged.

## Discussion

There is a spectrum of anomalies that can occur in the middle ear, including the ossicles. In mild cases, they can be the cause of conductive hearing loss, and in severe cases, it can cause cerebrospinal fluid (CSF) leakage. Dysplasia of the inner ear is often associated with an abnormal otic capsule, resulting in congenital weakness or fistula formation in the stapes footplate or annular ligament, which is one of the most common causes of cerebrospinal fluid otorrhea and meningitis (6). The present case had normal hearing prior to this episode, and the imaging showed an intact inner/middle ear structure. Intraoperatively, a minor anomaly of the stapes was identified, with a small membranous layer of tissue covering a bony defect in the center of the footplate. This type of congenital anomalous footplate has not been described. Recent developmental studies show that the possibility of this type of anomaly can still exist since the otic capsule may not be involved in the formation of the base of the stapes (7, 8). Because of her negative history of past traumatic events to her head or ears, this abnormal finding of the footplate is most probably due to developmental malformation.

Third window syndrome studies revealed that the intact bony labyrinth and differences in the stiffness of the oval and the round windows are essential for proper cochlear and vestibular function (9). Before her acute presentation to the hospital, this dehiscence in the footplate induced pathological pressure-evoked fluid-mechanical waves in the inner ear and caused pressure-induced vertigo, which is one of the TWS symptoms. Although she had vertigo induced by nose-blowing since she was 17 years old, the etiology remained undiagnosed. The rapid change in the middle ear/intracranial pressure by her nose-blowing resulted in membrane rupture, perilymph leakage, and pneumolabyrinth. When patients have susceptibility (e.g., weak structure) to rupture, such as that identified in this case, PLF can be caused by seemingly insignificant events such as nose-blowing.

The diagnosis has been established in CT+ TWS, which has typical symptoms and CT findings. Ward et al. synthesized the diagnostic criteria for SCD (10). On the other hand, the clinical entity of PLF with leakage has remained a topic of controversy for more than 50 years due to the lack of specific biomarkers. The manifestations of PLF with leakage include a broad spectrum of neuro-otological symptoms such as hearing loss, vertigo/dizziness, disequilibrium, aural fullness, tinnitus, and cognitive dysfunction. The hearing loss may range from high frequency to low frequency and can mimic Menière disease or cochlear endolymphatic hydrops. Therefore, the difficulty of making a definitive diagnosis of PLF has caused a long-standing debate regarding its prevalence, natural history, management, and even its very existence (11).

We can overcome this controversy if we could make the definite diagnosis of PLF with leakage using an appropriate biomarker. Based on proteomic analysis, we have identified an isoform of Cochlin CTP (12) as a perilymph-specific protein that is not expressed in the blood, CSF, or saliva (13). The leaked perilymph can be recovered by middle ear lavage (MEL) with 0.3 ml of saline. We have developed an ELISA for human CTP and defined the cutoff criteria as CTP ≧ 0.8 positive, 0.8 > CTP ≧ 0.4 intermediate, and 0.4 > CTP negative (ng/ml). The sensitivity and the specificity of the test to detect perilymph leakage was 86.4 and 100%, respectively (5). The detection of CTP in the middle ear indicates the presence of a fistula and perilymph leakage. The CTP test is the most extensively studied biomarker so far. In terms of perilymph-specific expression and diagnostic accuracy, a large-scale study has been reported (14). Using this novel test, the Japanese diagnostic criteria were established (Table 1). This test is continuously available as an investigator-initiated trial throughout Japan by Ikezono et al. (5) and funded by Saitama Medical University. In June 2020, the Japanese Ministry of Health Labor Standards approved the CTP ELISA Test, which has qualities for medical diagnosis.

**Table 1:** Diagnostic criteria for perilymph fistula (PLF) (based on the criteria of the Intractable Hearing Loss Research Committee of the Ministry of Health and Welfare, Japan revised in 2016).

**A. Symptoms**
Hearing impairment, tinnitus, aural fullness, and vestibular symptoms are observed in cases who had preceding events as listed below: (1) Coexisting or pre-existing middle and/or inner ear diseases (trauma, cholesteatoma, tumor, anomaly, SCCD, *etc*.), middle and/or inner ear surgeries (2) Barotrauma caused by antecedent events of external origin (e.g., blasting, diving, or flying, *etc*.) (3) Barotrauma caused by antecedent events of internal origin (e.g., nose-blowing, sneezing, straining, or carrying heavy objects, *etc*.)
**B. Laboratory findings**
(1) Microscopic/endoscopic inspection Visual identification of fistula(s) between the middle and the inner ear by a microscope or an endoscope. Fistulas can develop at the cochlear window, vestibular window, fracture site, microfissure, malformation, destruction in bony labyrinth caused by inflammation, *etc*. (2) Biochemical test Perilymph-specific protein is detected from the middle ear
**C. Reference**
(1) A perilymph-specific protein; e.g., Cochlin-tomoprotein (CTP) detection test. After myringotomy, the middle ear is rinsed with 0.3 ml saline three times; the fluid was recovered (middle ear lavage, MEL) and tested by polyclonal antibody ELISA. The cutoff criteria: ~0.4 < CTP-negative; 0.4 ≦ CTP < 0.8 intermediate; 0.8 ≦ CTP-positive (2) Idiopathic cases may exist (3) The following symptoms and/or test results may be observed: 1. Streaming water-like tinnitus or feeling of running water in the middle ear 2. A popping sound can be heard at the onset 3. Nystagmus and/or vertigo induced by pressure application to the middle ear (Hennebert's phenomenon, fistula sign) 4. Imaging studies may show a fistula in the bony labyrinth or pneumolabyrinth 5. Progression of hearing impairment, tinnitus, and aural fullness may be acute, progressive, fluctuating, or recurrent 6. The main complaints can be vestibular symptoms without hearing impairment
**D. Differential diagnosis**
Inner ear diseases with known causes, such as viral infection, genetic, vestibular schwannoma, *etc*.
**E. Diagnosis**
Probable PLF: only symptoms listed in ADefinite PLF: symptoms and laboratory findings listed in B

Perilymph leakage may be located in the round or the oval window (15), which may be associated with an anomalous stapes footplate, such as in this case. We have also reported a case with patent fistula ante-fentestram, showing that this microfissure can be the route for perilymph leakage (16). Most pneumolabyrinth patients reported to date were due to temporal bone trauma or otologic surgery (17–19). Pneumolabyrinth was shown by CT imaging in this case, which has a strong diagnostic value for perilymph leakage induced by the barotraumatic event. Imaging by high-resolution temporal bone CT did not show a CT+ TWS or any other inner ear abnormalities, suggesting that the pressure-induced vertigo that she experienced before her acute presentation was due to bony dehiscence of the center of the stapes footplate.

This case demonstrates that PLF is a real clinical entity. It is noteworthy that, unlike other causes of sensorineural hearing loss and dizziness, PLF with leakage is surgically correctable by sealing the fistula. By sealing the fistula, PLF is a surgically curable disease. Also, appropriate recognition and treatment of PLF can improve a patient's condition and, hence, quality of life.

## Data Availability Statement

The raw data supporting the conclusions of this article will be made available by the authors, without undue reservation.

## Ethics Statement

The studies involving human participants were reviewed and approved by Institutional Review Board of Saitama Medical University Hospital (IRB No.13086). The patients/participants provided their written informed consent to participate in this study. Written informed consent was obtained from the individual for the publication of any potentially identifiable images or data included in this article.

## Author Contributions

TI and HM: data curation and conceptualization. TM: formal analysis. TI, S-iU, and YK: funding acquisition. HM, YT, TS, TM, SSa, SSh, YK, AI, and TI: investigation. TI, TM, SSa, and HM: methodology. TI: project administration, resources, supervision, validation, and visualization. HM: writing–original draft. All authors contributed to the article and approved the submitted version.

## Conflict of Interest

TI has a patent on this CTP detection test. The remaining authors declare that the research was conducted in the absence of any commercial or financial relationships that could be construed as a potential conflict of interest.
